# P-257. Performance of Serum Cryptococcal Antigen for Early Detection of Cryptococcal Meningitis in People Living with HIV

**DOI:** 10.1093/ofid/ofaf695.478

**Published:** 2026-01-11

**Authors:** Victor D Acuña-Rocha, Gabriel G Ibarra-Nuñez, Adrian Camacho-Ortiz, Paola Bocanegra-Ibarias, Samantha Flores-Treviño, Daniel Salas-Treviño, Eduardo Pérez-Alba, Laura Nuzzolo-Shihadeh

**Affiliations:** Servicio de Infectología, Hospital Universitario “Dr. José Eleuterio González”, Monterrey, Nuevo Leon, Mexico; Departamento de Medicina Interna, Hospital Universitario “Dr. José Eleuterio González”, Universidad Autónoma de Nuevo León, Monterrey, Nuevo Leon, Mexico; Universidad Autónoma de Nuevo León, Monterrey, Nuevo Leon, Mexico; Servicio de Infectologia - Hospital Universitario " Dr. Jose Eleuterio Gonzalez", Monterrey, Nuevo Leon, Mexico; Servicio de Infectología, Hospital Universitario “Dr. José Eleuterio González”, Monterrey, Nuevo Leon, Mexico; Servicio de Infectología, Hospital Universitario “Dr. José Eleuterio González”, Monterrey, Nuevo Leon, Mexico; Servicio de Infectología, Hospital Universitario “Dr. José Eleuterio González”, Monterrey, Nuevo Leon, Mexico; Servicio de Infectología, Hospital Universitario “Dr. José Eleuterio González”, Monterrey, Nuevo Leon, Mexico

## Abstract

**Background:**

The development of cryptococcal antigen (CrAg) detection methods, such as latex agglutination, ELISA, and lateral flow assays has significantly improved early diagnosis, achieving sensitivities and specificities between 99.1% and 100% (1-4). This study aimed to assess the utility of serum CrAg (SCrAg) screening and subsequent early treatment in reducing in-hospital mortality among people living with HIV (PLHIV) in a teaching hospital.

**Figure:**
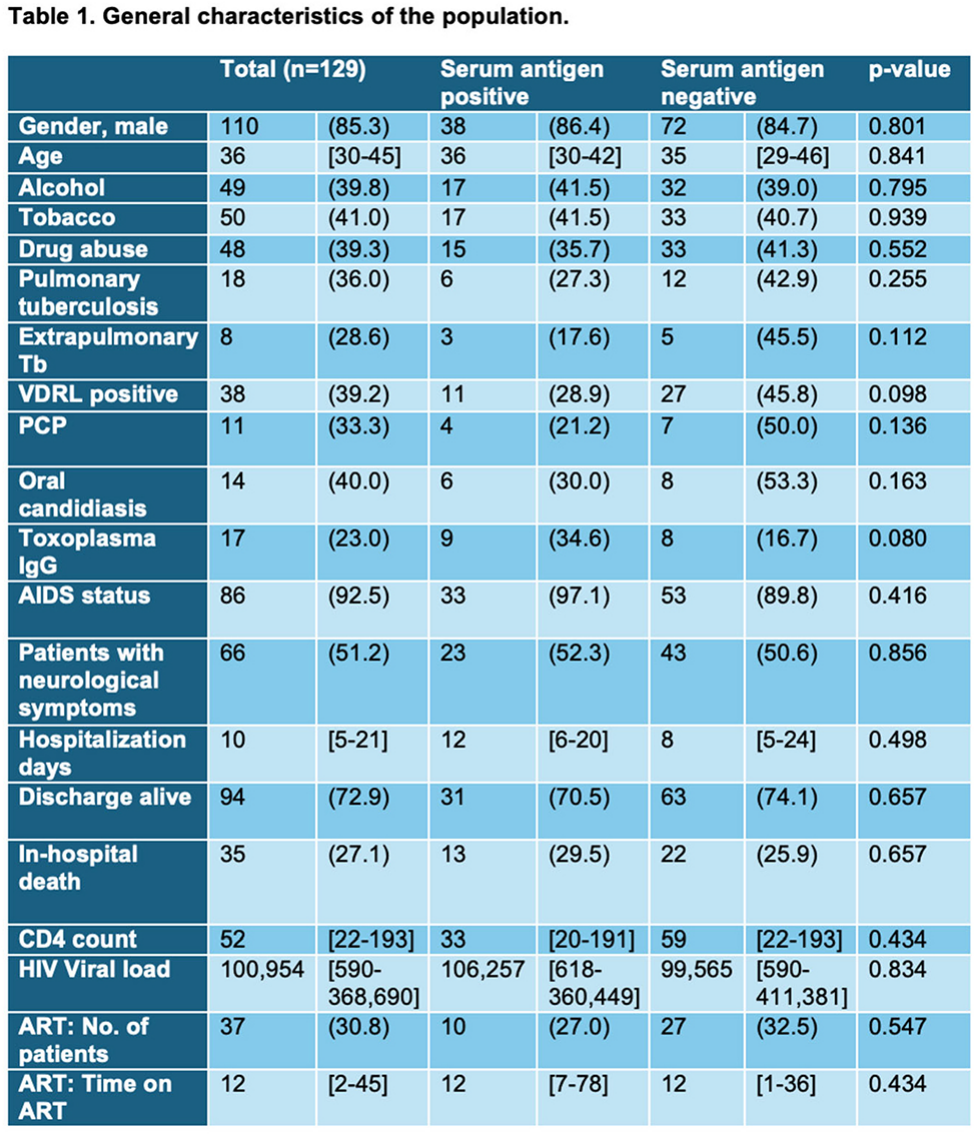

**Figure:**
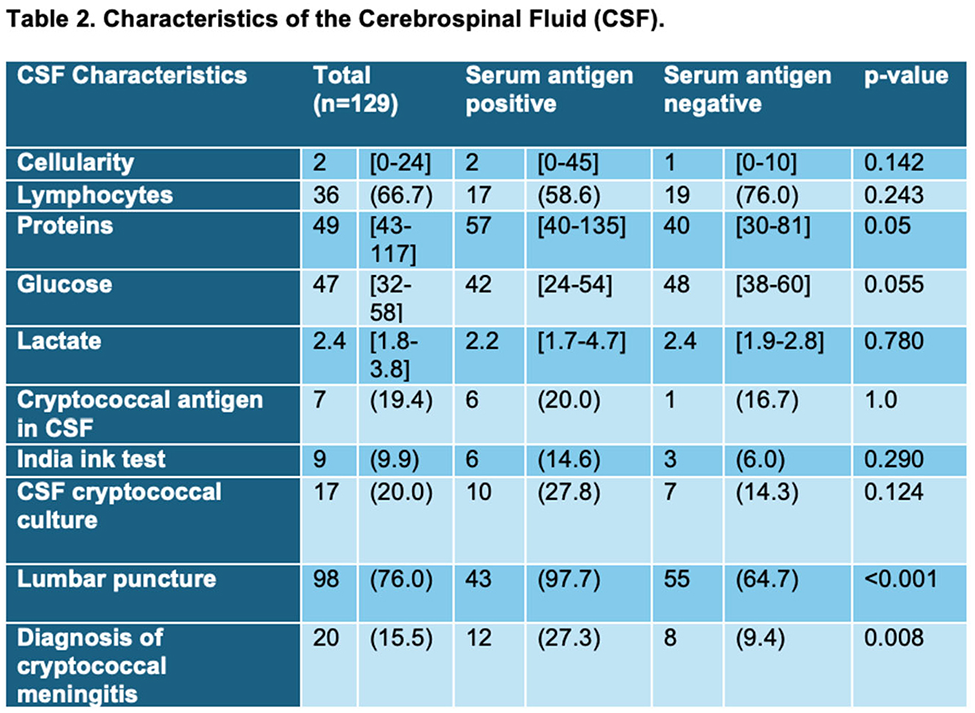

**Methods:**

We conducted a descriptive, retrospective, cross-sectional, and analytical study of adults (≥18 years) living with HIV who were hospitalized in clinical stage C for any reason between December 2021 and June 2023 at the University Hospital “Dr. José Eleuterio González.” Clinical and laboratory data were collected from hospital records. Qualitative serum and CSF cryptococcal antigen (CrAg) were detected using the Cryptococcal Antigen Latex Agglutination System (Meridian Bioscience®). All patients with a positive serum CrAg (SCrAg) result underwent lumbar puncture for cytochemical analysis, India ink staining, microbiological culture, and CSF CrAg testing.

**Figure:**
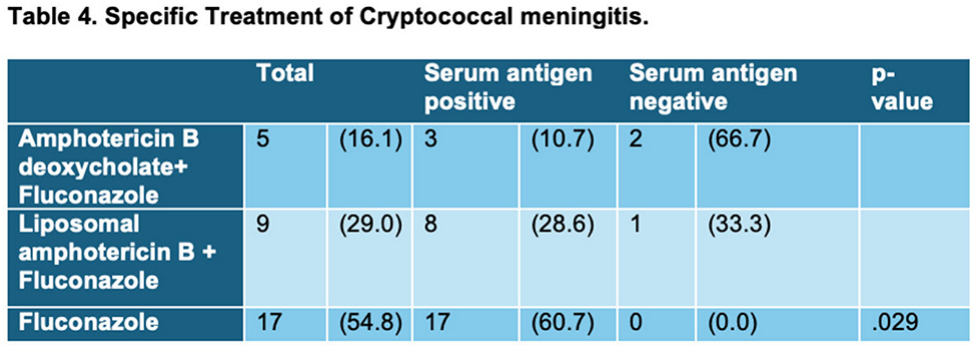

**Results:**

A total of 129 people living with HIV (PLWHIV) were included in the study, of whom 85.3% (n=110) were male at birth. The median age was 36 years [30-45]. Among the 129 PLWHIV, 34.1% (n=44) tested positive for serum cryptococcal antigen. In contrast, 65.9% (n=85) PLWHIV tested negative for serum cryptococcal antigen.

Overall, CSF CrAg was positive in 19.4% of patients (n=7); this included 20% (n=6) of those with a positive serum CrAg (SCrAg) and 16.7% (n=1) of those with a negative SCrAg. India ink staining was positive in 9.9% (n=9) of the patients, and cryptococcal cultures in CSF were positive in 20.0% (n=17), with no difference in the antigen-positive group, 27.8% (n=10) vs the antigen-negative group, 14.3% (n=7) (p=0.124).

Cryptococcal meningitis was diagnosed in 15.5% (n = 20) of patients, occurring more often in the serum antigen-positive group (27.3%, n = 12) compared to the serum antigen-negative group (9.4%, n = 8; p = 0.008).

**Conclusion:**

Universal SCrAg screening in hospitalized PLWHIV increased lumbar puncture rates and cryptococcal meningitis diagnoses. However, despite early treatment, our results did not show a difference in hospital mortality.

**Disclosures:**

All Authors: No reported disclosures

